# Near-Infrared-Triggered Photodynamic Therapy toward Breast Cancer Cells Using Dendrimer-Functionalized Upconversion Nanoparticles

**DOI:** 10.3390/nano7090269

**Published:** 2017-09-11

**Authors:** Bing-Yen Wang, Ming-Liang Liao, Guan-Ci Hong, Wen-Wei Chang, Chih-Chien Chu

**Affiliations:** 1 Division of Thoracic Surgery, Department of Surgery, Changhua Christian Hospital, Changhua County 50006, Taiwan; 156283@cch.org.tw; 2 School of Medicine, Chung Shan Medical University, Taichung City 40201, Taiwan; 3 Institute of Genomics and Bioinformatics, National Chung Hsing University, Taichung City 40227, Taiwan; 4 School of Medicine, Kaohsiung Medical University, Kaohsiung City 80708, Taiwan; 5 Department of Medical Applied Chemistry, Chung Shan Medical University, Taichung City 40201, Taiwan; withoutyou@hotmail.com.tw; 6 Department of Biomedical Sciences, Chung Shan Medical University, Taichung City 40201, Taiwan; gqttw981@mail.ntust.edu.tw; 7 Department of Medical Education, Chung Shan Medical University Hospital, Taichung City 40201, Taiwan

**Keywords:** upconversion, dendrimers, photodynamic therapy, layer-by-layer, cancer stem cells, near-infrared, tumorspheres

## Abstract

Water-soluble upconversion nanoparticles (UCNPs) that exhibit significant ultraviolet, blue, and red emissions under 980-nm laser excitation were successfully synthesized for performing near infrared (NIR)-triggered photodynamic therapy (PDT). The lanthanide-doped UCNPs bearing oleate ligands were first exchanged by citrates to generate polyanionic surfaces and then sequentially encapsulated with NH_2_-terminated poly(amido amine) (PAMAM) dendrimers (G4) and chlorine6 (Ce6) using a layer-by-layer (LBL) absorption strategy. Transmission electron microscopy and X-ray diffraction analysis confirm that the hybrid UCNPs possess a polygonal morphology with an average dimension of 16.0 ± 2.1 nm and α-phase crystallinity. A simple calculation derived through thermogravimetric analysis revealed that one polycationic G4 dendrimer could be firmly accommodated by approximately 150 polyanionic citrates through multivalent interactions. Moreover, zeta potential measurements indicated that the LBL fabrication results in the hybrid nanoparticles with positively charged surfaces originated from these dendrimers, which assist the cellular uptake in biological specimens. The cytotoxic singlet oxygen based on the photosensitization of the adsorbed Ce6 through the upconversion emissions can be readily accumulated by increasing the irradiation time of the incident lasers. Compared with that of 660-nm lasers, NIR-laser excitation exhibits optimized in vitro PDT effects toward human breast cancer MCF-7 cells cultured in the tumorspheres, and less than 40% of cells survived under a low Ce6 dosage of 2.5 × 10^−7^ M. Fluorescence microscopy analysis indicated that the NIR-driven PDT causes more effective destruction of the cells located inside spheres that exhibit significant cancer stem cell or progenitor cell properties. Moreover, an in vivo assessment based on immunohistochemical analysis for a 4T1 tumor-bearing mouse model confirmed the effective inhibition of cancer cell proliferation through cellular DNA damage by the expression of Ki67 and γH2AX^ser139^ protein markers. Thus, the hybrid UCNPs are a promising NIR-triggered PDT module for cancer treatment.

## 1. Introduction

Recently, the biomedical applications of lanthanide-doped upconversion nanoparticles (UCNPs) have attracted much attention [1,2,3,4,5,6]. Because UCNP-based materials can sequentially absorb two or more photons, they exhibit a unique anti-Stokes shift of fluorescence emission in ultraviolet (UV)-visible wavelengths (300–700 nm) under NIR light excitation (750–1400 nm). The unique “photon upconversion” process has several merits, including reduced fluorescence background, improved tissue penetration, and lower phototoxicity in biological fields [7]. Moreover, UCNPs usually exhibit multiple fluorescence emission bands of UV, blue-green, and red light simultaneously under NIR excitation. Additionally, finely manipulating the doped-metal composition or the host crystal structure can lead to specific upconverted emissive patterns. For example, the nanoparticles doped with erbium (Er^3+^) instead of thulium (Tm^3+^) can switch emission wavelengths from blue to green light as well as enhance red-light intensity; a single band, intense red-light emission is achieved by incorporating manganese (Mn^2+^), ytterbium (Yb^3+^), and Er^3+^ into the nanostructure [8,9,10]. Combined with the robust, well-developed synthetic protocols, UCNPs bearing specific upconverted emission profiles are promising for photo-assisted cancer treatments, particularly photodynamic therapy (PDT) [11,12,13,14].

PDT based on the photochemical reactions of photosensitizers (PSs) is a treatment strategy involving light irradiation at appropriate wavelengths, by which PS molecules can produce cytotoxic reactive oxygen species (ROS) and singlet oxygen (^1^O_2_) to destroy nearby cancer cells [15]. The greatest advantage of PDT is the ability to selectively treat tumor cells by using light under spatiotemporal control. However, most PS molecules are excited by visible or UV light, which have poor tissue penetration, thus limiting the treatment of large or internal tumors. Loading the PS molecules onto the UCNPs surface avoids this drawback, as it facilitates NIR-triggered PDT, which is based on upconverted fluorescence emission and resonance energy transfer. Several strategies have been developed to immobilize the PS molecules on the surface including mesoporous silica coating and polymer encapsulation [9,16,17,18,19]. Intense upconverted emission at suitable wavelengths and higher loading capacity of the active molecules was proposed for guaranteeing more effective therapeutic outcomes. Liu and coworkers demonstrated a layer-by-layer (LBL) method for loading multiple layers of PS-conjugated polymers onto the surface of UCNPs surface through electrostatic interaction [13]. Both in vitro and in vivo PDT effects were substantially improved through their multiloading strategy. As clinical PDT develops, fabricating PS-conjugated-UCNP systems with commercialization potential by using more facile and robust synthetic protocols remains challenging.

**Scheme 1:**
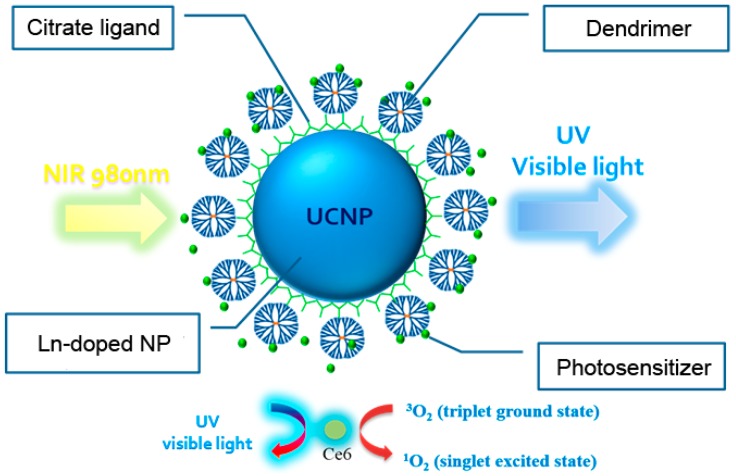
Near-infrared (NIR)-light-triggered photodynamic therapy based on a lanthanide (Ln)-doped NaYF_4_ upconversion nanoparticle (UCNP) sequentially encapsulated with citrates, PAMAM dendrimers, and chlorin e6 (Ce6) via a layer-by-layer (LBL) absorption method.

This paper introduces the application of dendrimers in the encapsulation layers of UCNPs for trapping PS molecules according to the multivalency of the dendritic structures [20]. Dendrimers, well-defined hyperbranched polymers consisting of a central core, radiating branches ending in multivalent terminal groups, and cavities within the branched structure have aroused much interest in the past two decades [21,22,23,24]. For example, full-generation poly(amido amine) (PAMAM) dendrimers bear multiple primary amines on the surface and internal tertiary amines as branching sites. These tertiary amines can be protonated to generate polycations at physiological pH, thus allowing for electrostatic interaction and subsequent formation of complexes with molecules bearing opposite charges [25,26,27,28]. Apart from electrostatic binding, the internal cavities within the dendrimers can accommodate small molecules through host-guest affinity. Accordingly, typical fourth-generation PAMAM dendrimers (G4), bearing 64 peripheral amines, were immobilized on the surface by using the facile LBL strategy to create positively charged UCNPs. The material design enhanced the cellular uptake of the nanoparticles as well as the absorption of negatively charged PS molecules by those dendrimers (Scheme 1). A 980-nm NIR laser diode (LD) was used as the light excitation source to analyze upconversion and PDT effect. To elucidate the NIR-triggered PDT efficacy, we explore the ^1^O_2_ production combined with the apoptosis of breast cancer cells cultured in two-dimensional (2D) arrays and three-dimensional (3D) spheres and with the cellular DNA damage of tumor tissues [29,30].

## 2. Results and Discussion

### 2.1. Preparation of Lanthanide-Doped UCNPs

**Figure 1:**
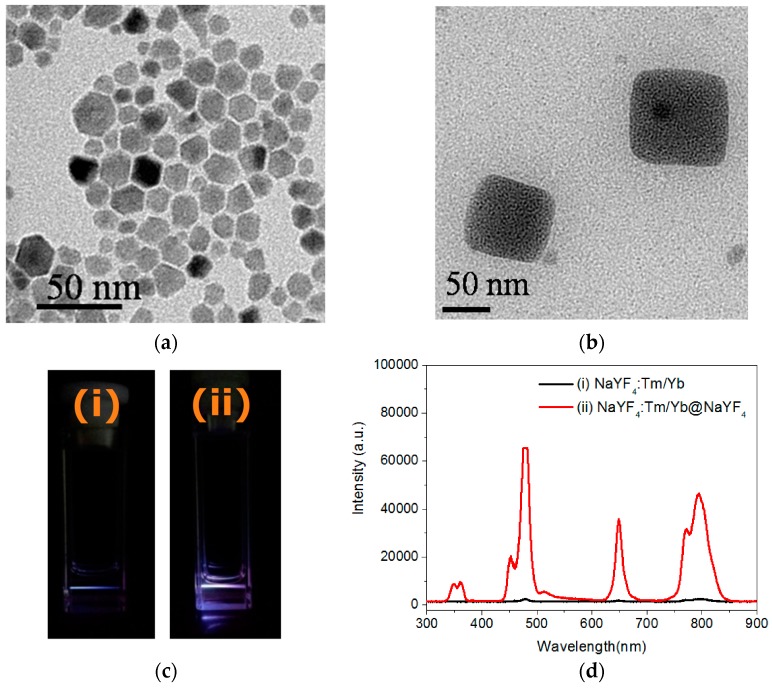
Transmittance electron microscopic (TEM) images of: (**a**) polygonal (small); and (**b**) cubic-like (large) NaYF_4_ nanocrystals. The average dimension for (**a**) was calculated based on 100 nanoparticles selected from a TEM micrograph. Upconversion fluorescence images (**c**) and spectra (**d**) of the: (i) core; and (ii) core-shell nanocrystals, under 980-nm laser excitation. The spectra recorded by a CCD imaging spectrometer exhibits UV, blue, red, and NIR wavelengths simultaneously.

**Figure 2:**
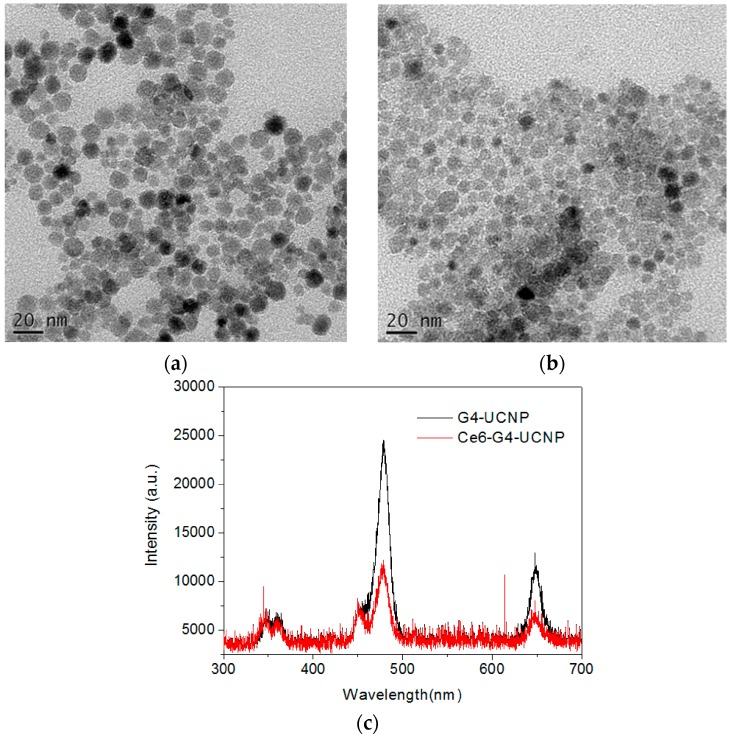
Transmittance electron microscopic (TEM) images of: (**a**) citrate; and (**b**) PAMAM dendrimer-encapsulated nanoparticles (G4-UCNP). (**c**) Upconversion fluorescence of G4-UCNP in water under 980-nm laser excitation. The fluorescence intensity was extinguished after encapsulation of Ce6 (Ce6-G4-UCNP).

Yb^3+^- and Tm^3+^-doped NaYF_4_ nanocrystals were used as core materials in the NIR-induced PDT because of their high efficacy in NIR-to-visible light upconversions. Herein, chlorin e6 (Ce6), a chlorophyll analog with significant absorption, maximum at approximately 400 nm (Soret band) and 660 nm (Q-band), was used as the PS in the PDT investigation at the cellular level [31,32]. The UCNPs were synthesized using a solvothermal procedure at 300 °C with oleic acid (OA) as the stabilizer [33]. To enhance the energy transfer between the doped ions and host materials, the core of the UCNPs was further covered by a layer of undoped NaYF_4_ to form a core-shell [34]. The crystallinity and composition for the UCNPs were characterized using X-ray diffraction (XRD) equipped with energy dispersive X-ray spectroscopy (EDXS), suggesting α-phase (cubic-like) NaYF_4_ with successful Yb^3+^ and Tm^3+^ doping (Supplementary Materials, Figures S1 and S2). As shown in Figure 1a, the images of the core-shell nanocrystals, taken using TEM, display a polygonal morphology with an average dimension of 16.0 ± 2.1 nm. Moreover, a cubic-like nanocrystal larger than 50 nm was clearly observed in certain grid areas, which is consistent with the XRD data (Figure 1b). As shown in Figure 2c, the prepared UCNPs emitted upconverted fluorescence at multiple bands of UV (λ_max_ = 360 nm), blue (λ_max_ = 480 nm), red (λ_max_ = 650 nm), and NIR (λ_max_ = 795 nm) wavelengths under 980 nm LD excitation. Notably, the fluorescence intensity was considerably increased by the core-shell strategy. Among the bands, the UV and red emissions are capable of exciting the Ce6 that possesses corresponding absorption, and more intense blue and NIR emissions allow for tracing the biodistribution of the UCNPs through fluorescence microscopy.

### 2.2. Fabrication of UCNPs through Ligand Exchange and the LBL Strategy

Synthetic strategies involving the encapsulation of mesoporous silica and water-soluble polymers have been developed for preparing the hydrophilic UCNPs used in biomedicinal applications. To fabricate the nanoparticle surface with polycationic G4 in this hybrid system, negatively charged UCNPs are produced and, the dendrimers are electrostatically adsorbed using the LBL method [13]. The first attempt of using an ozonolysis reaction to cleave the double bonds of the oleate ligands for generating anionic carboxylate groups was unsuccessful, because the oxidative treatment under acidic condition led to the unexpected desorption of the surface ligands [35]. The removal of the ligands was confirmed by thermogravimetric analysis (TGA) measurement (Supplementary Materials, Figure S3) [12]. Alternatively, the oleate ligands were readily exchanged by a trivalent ligand of sodium citrate in diethylene glycol under 200 °C, thus allowing for the fabrication of the UCNPs with multiple carboxylate groups as the first layer [36]. The NH_2_-determinated G4 dendrimers on the UCNPs were then immobilized through electrostatic interaction at ambient temperature, thus generating water-dispersible nanoparticles with excess positive charges on the surface and forming the second layer. After the exchange with the citrate ligands, the zeta potentials of the modified UCNPs revealed a negatively charged surface (ζ = −8.54 mV), however, a positive charge (ζ = +31.7 mV) presented after the dendrimer encapsulation (Supplementary Materials, Figure S4). Moreover, FT-IR analysis confirmed the exchanged citrate ligands through the characteristic carboxylate stretching at 1586 cm^−1^and 1400 cm^−1^, and the featured amide stretching of the PAMAM dendrimers adsorbed on the surface was well characterized at 1640 cm^−1^and 1560 cm^−1^ (Supplementary Materials, Figure S5). As illustrated by the TEM images shown in Figure 2a,b, the morphology and dimensions of the UCNPs were mostly unaltered after citrate and dendrimer modification, but the boundary of each dendrimer-encapsulated nanoparticle was less defined. This observation was presumably because of the strengthened interactions between the nanoparticles including the hydrogen bonding and van der Waals force produced by the G4 PAMAM dendrimers. Moreover, the hydrodynamic dimension of the G4-functionalized UCNPs determined by dynamic light scattering is approximately 180 nm with a narrow distribution, suggesting that these dendrimers were successfully immobilized on the surfaces of the UCNPs (Supplementary Materials, Figure S6). Although the dendrimer interaction may have led to substantial aggregation in these water-dispersible UCNPs, the upconversion emission still exhibited distinct fluorescence bands in the UV-, blue-, and red-light regions (Figure 2c).

The organic contents of the modified UCNPs were calculated using the weight-loss data obtained through TGA, and approximately 15% and 25% of the oleate and citrate ligands, respectively, were immobilized on the UCNP surface (Supplementary Materials, Figure S3). Assuming that: (1) every UCNP exhibited a spherical shape and uniform size upon TEM analysis; and (2) the mass of a nanoparticle is calculated from the bulk density of NaYF_4_ (4.21 g/cm^3^), the ligand density per particle can be determined using the simple calculation proposed by Winnik and coworkers [37]. The coverage densities of oleate- and citrate-modified UCNPs, were 4.8 and 11 ligands/nm^2^, respectively; thus, approximately 4000 oleate and 9000 citrate molecules covered each nanoparticle. Moreover, by finely adjusting the feeding concentration of dendrimers to UCNPs, approximately 60 dendrimers were delivered to one citrate-modified UCNP. Based on Winnik and coworkers’ calculation, one polycationic G4 dendrimer was theoretically accommodated by 150 polyanioinic citrate molecules. Accordingly, the dendrimers could be firmly immobilized on the UCNP surface through multivalent interactions.

**Figure 3:**
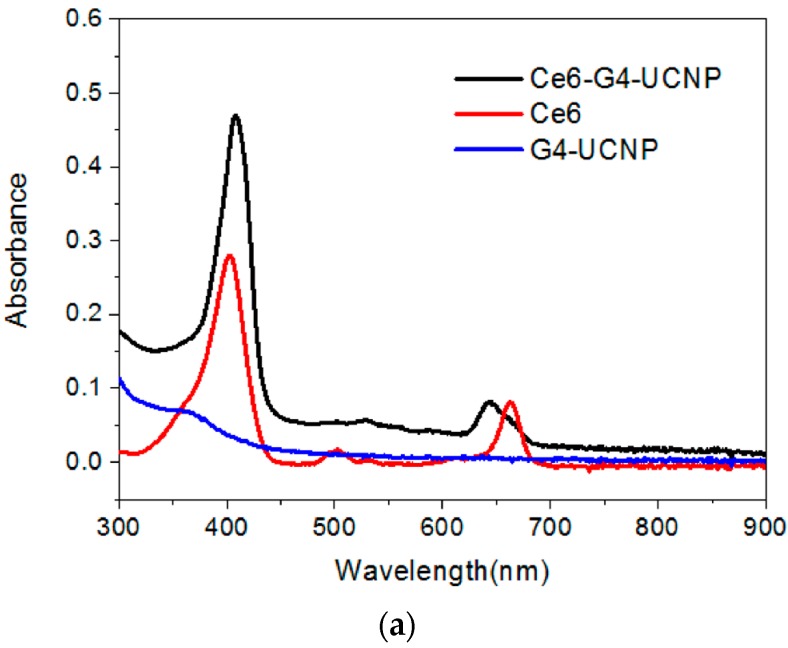

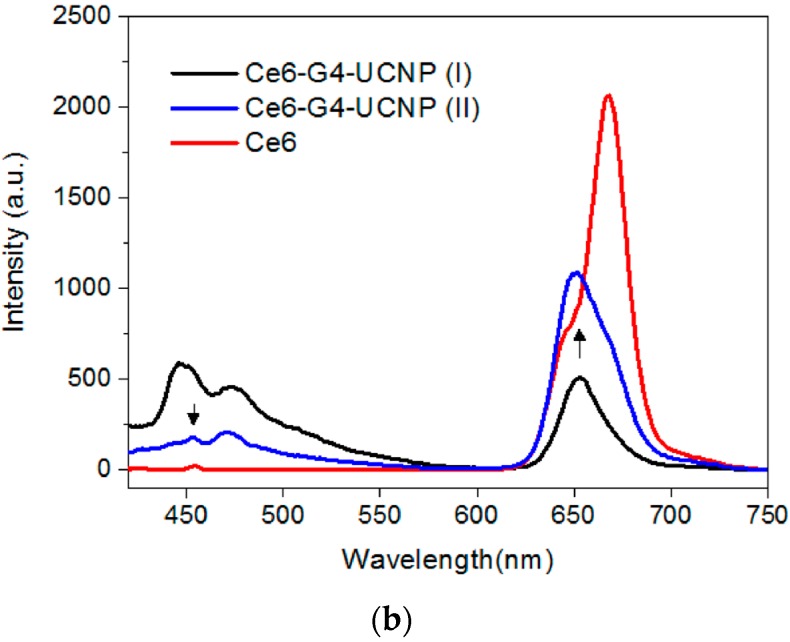
(**a**) UV-Vis absorption; and (**b**) fluorescence spectra of the hybrid nanoparticles sequentially encapsulated with dendrimers (G4-UCNP) and Ce6 (Ce6-G4-UCNP) in comparison with that of pristine Ce6; (I) and (II) denote different loading capacity of Ce6.

The negatively charged Ce6 molecules were then directly loaded onto the dendrimer-encapsulated UCNPs to form the third layer through the electrostatic binding of the COOH and NH_2_ groups. The zeta potential of the hybrid UCNPs decreased from +31.7 mV to +24.0 mV, suggesting that negatively charged Ce6 molecules covered the surface of the nanoparticles and thus partially neutralized the cationic density produced by the dendrimers (Supplementary Materials, Figure S4). Moreover, as shown in Figure 3a, the UV-Vis spectrum also confirmed the Ce6 adsorption through the characteristic Soret and Q-band absorption bands at 407 nm and 643 nm, respectively. Notably, the Q-band of the hybrid UCNPs exhibited substantial blue-shifted wavelength compared with that of free Ce6, which suggests that the PS molecules were entrapped by the surface dendrimers. Generally, the interior cavities of PAMAM dendrimers possess a hydrophobic domain. Once chromophores or fluorophores are trapped inside the dendritic cavities, their absorption or emission wavelength shifts hypsochromically in contrast to the free probe molecule in aqueous solution [38]. Therefore, the red fluorescence emission of the water-immersed hybrid UCNPs under the excitation of the Soret band was also blue shifted by approximately 20 nm (Figure 3b). In addition, the loading capacity of the Ce6 molecules could be readily determined based on the fluorescence intensity of the non-adsorbed Ce6 that washed off the hybrid UCNPs. The loading amount increased with the initial feeding concentration of the Ce6. This was due to the multivalent character of the dendrimers, assisting the adsorption of the PS molecules through the exterior and interior interactions caused by electrostatic and host-guest affinity, respectively. Figure 3b also shows a blue-to-green fluorescence band (430–580 nm), which is presumably attributable to the intrinsic emission of the G4 dendrimer [28,39,40]. Notably, the fluorescence intensity of the G4 decreased with increasing Ce6 content. This result implies a strong interaction between the dendrimer and the entrapped Ce6, because the emission of the dendrimer and the absorption of the Ce6 occupied proximal wavelength regions, thus facilitating the fluorescence energy transfer. Moreover, this result clearly indicates that the PS molecules were firmly immobilized by the PAMAM dendrimers on the surfaces of the UCNPs. Figure 2c depicts the upconversion fluorescence spectra of the aqueous hybrid UCNPs under NIR-laser excitation. The emission wavelengths centered at the UV-, blue-, and red-light regions were unaltered after the surface modification. However, the visible-light emissions of the UCNPs were extinguished by the peripheral Ce6, principally through the Soret and Q-band absorptions, a manifestation of effective energy transfer from the UCNPs to the surrounding PS molecules [41,42,43]. Notably, even the weak absorption band of the Ce6 (Figure 3a) at approximately 480–520 nm demonstrated substantial absorption of the blue fluorescence emitted from the UCNPs. Finally, the PS molecules were successfully photoexcited by the 980-nm NIR laser, using the dendrimer-encapsulated UCNPs as the nanoplatform.

### 2.3. The Evaluation of Photoactivity and Singlet Oxygen (^1^O_2_) Formation

**Figure 4:**
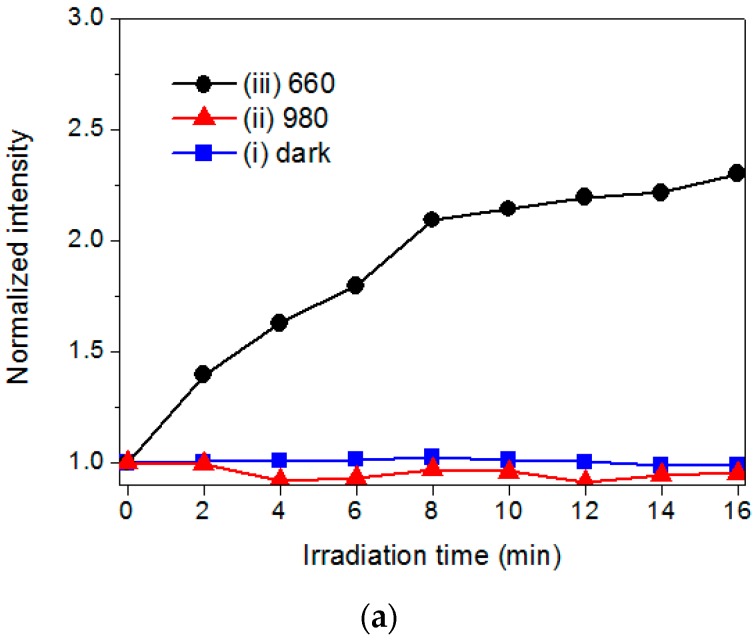

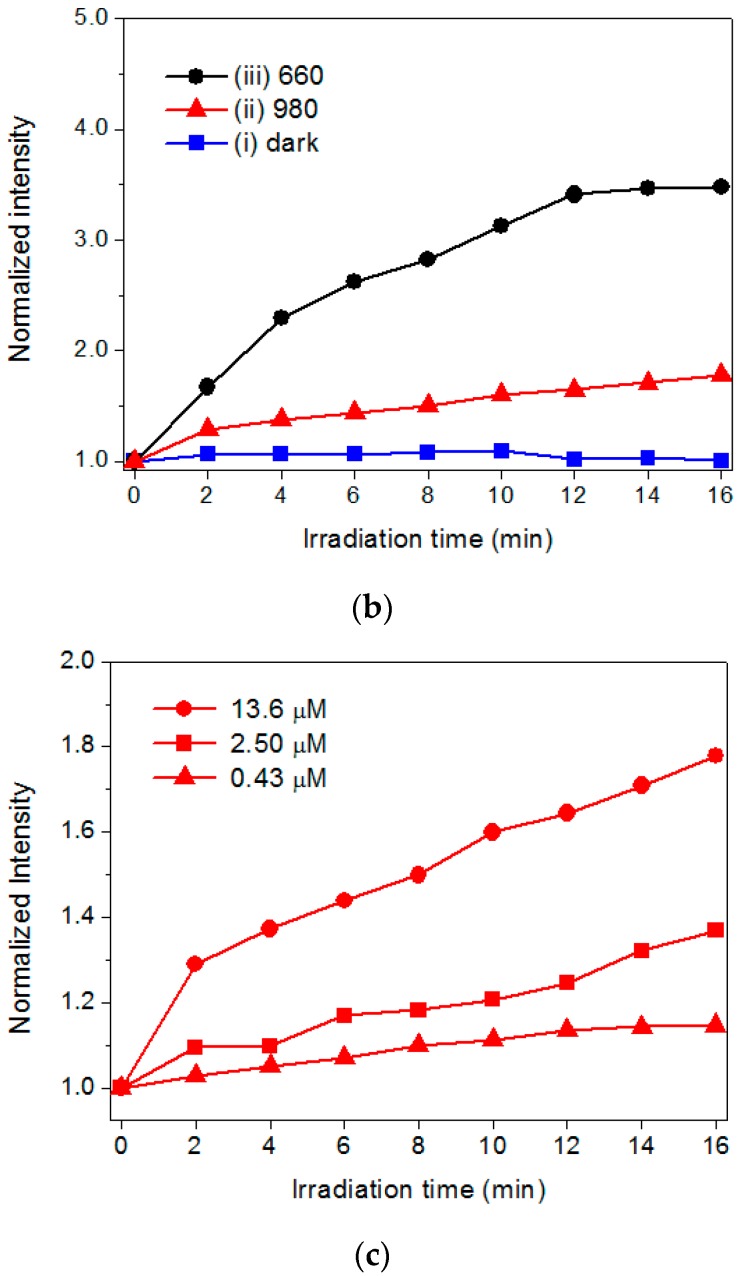
The FCLA assay for determining the accumulation of singlet oxygen using: (**a**) pristine Ce6; and (**b**) hybrid nanoparticles (Ce6-G4-UCNP) as the photosensitizer: (i) dark condition; (ii) 980; and (iii) 660-nm laser excitation; and (**c**) the Ce6-G4-UCNP with increasing loading capacity of Ce6 (0.43, 2.50, and 13.6 μM) under 980-nm laser excitation. The fluorescence intensity was normalized to the intrinsic fluorescence of FCLA solution in the absence of additives and light exposure.

To assess the capability of the hybrid UCNPs to generate ^1^O_2_, a fluoresceinyl Cypridina luciferin analog (FCLA) was employed as a chemiluminescence probe [14]. The FCLA was oxidized by ^1^O_2_ and thus markedly enhanced fluorescence by 524 nm. Figure 4a,b illustrates the correlation of the laser irradiation time and fluorescence intensities of FCLA in the presence of PSs. The Ce6 and hybrid UCNPs exhibited fluorescence intensities of approximately 2.3-fold and 3.5-fold increments, respectively, and plateaued upon 660-nm laser irradiation for 16 min, by which the Ce6 molecules were directly photoexcited through the Q-band absorption. The FCLA assay could detect the accumulation of the ^1^O_2_ in water by increasing the exposure time of the red light. Notably, the hybrid UCNPs were also sensitive to the irradiation using a 980-nm NIR laser, displaying a two-fold increase in fluorescence; whereas, the Ce6 was completely unreactive to the NIR light. This result clearly confirmed that the Ce6 immobilized by the dendrimers was photoexcited by the upconverted fluorescence emitted from the UCNPs nanoplatform. Moreover, as shown in Figure 4c, the UCNPs with a higher loading of Ce6 generated substantial ^1^O_2_ under 980-nm laser irradiation, attributed to the merits of using the polyvalent dendrimers as the adsorption layer to accommodate more PS molecules through both exterior and interior interactions. Considering all the assay data, the photoactivation permitted energy transfer between the excited Ce6 and surrounding oxygen molecules, generating significant ^1^O_2_ as the major ROS in NIR-light-triggered PDT for solid tumors.

### 2.4. In Vitro PDT Analysis for Human-Breast Cancer Cells

Laser-triggered PDT was performed on the MCF-7 cell lines that were cultured in the 2D and 3D models [44,45]. Generally, 2D cell-culture systems are a convenient way to study cancer cells in vitro; however, tissue-specific architecture with cell–cell and cell-extracellular matrix interactions are reduced when cells grow on flat and adherent substrates. Solid tumors growing in a 3D spatial conformation are currently being developed as more advanced in vitro models. A key advantage of 3D cultures in preclinical research is their capacity to modulate the molecular gradients that exist in living tissues, such as oxygen, nutrients, metabolites, and signaling molecules. Hence, 3D models mimic the complexity of tissues more precisely than conventional 2D monolayers, thus potentially bridging the gap between 2D cultures and animal models. Moreover, cells cultured in a 3D scaffold generate a cell population by enhancing the properties of cancer stem cells (CSCs), which have been demonstrated to retain cancer-initiating potential and self-renewal capability and lead to tumorigenesis and drug resistance in malignant tumors in vivo. In addition, 3D cell cultures usually upregulate the expression of hypoxia-responsive genes and are thus more resistant to PDT than 2D monolayers, because of limited oxygen supply in the 3D spheroids. Accordingly, performing NIR-triggered PDT in a 3D model of the MCF-7 CSCs using the hybrid UCNPs as the PS is a therapeutic challenge.

Prior to PDT analysis, the control experiment shows that 660-nm or 980-nm laser irradiation has no influence on the survival of MCF-7 cell lines. Under darkness, Ce6 was nontoxic for the cells cultured in a conventional 2D system at a high dose (10 μM), but it immediately became a chemotherapy drug and completely destroyed target cells under 660-nm laser excitation for 10 min, which potentially favored photodynamic medication under spatiotemporal control. However, the PDT outcome was ineffective when the 980-nm NIR laser was used as the excitation source, because the Ce6 was nearly transparent at this wavelength and photoinduced ^1^O_2_ production was inhibited. The median lethal dose of Ce6 for the MCF-7 cells under 660-nm laser irradiation was below 1 μM, suggesting that Ce6 is an effective PS responsive to the red light.

**Figure 5:**
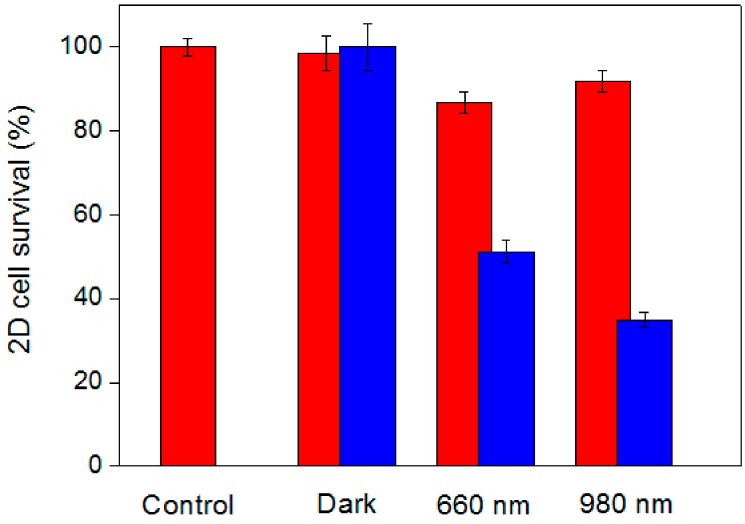
Photo-induced cytotoxicity for MCF-7 cells cultured in a 2D system in the presence of pristine Ce6 (red) and hybrid nanoparticles (blue) as the photosensitizer under laser irradiation. [Ce6] = 0.25 μM.

Figure 5 presents the survival test results for the 2D cell cultures, using the hybrid UCNPs as the PS under laser excitation. Herein, the loading concentration of the Ce6 on the UCNP determined through fluorescence spectroscopy was approximately 0.25 μM. The control experiments demonstrated that the hybrid UCNPs were also nontoxic in the dark and that 86% and 91% of the MCF-7 cells survived in the presence of pristine Ce6 under 660-nm and 980-nm laser irradiation, respectively. This result suggests that the PDT efficiency was quite low at this Ce6 dosage under laser excitation. In sharp contrast, when the Ce6 was loaded onto the dendrimer-modified UCNPs, approximately 50% of the cells died under 660-nm light. Compared with the Ce6, the hybrid UCNPs exhibited higher PDT performance, presumably because of higher cellular-uptake efficiency through endocytosis. Generally, the accumulation of Ce6 in target cells is inefficient because the negatively charged –COOH groups may be electrostatically repulsed by the cell membranes; based on the surface PAMAM dendrimers, the hybrid UCNPs possess multiple positive charges, thus favoring the cellular uptake of the nanoparticles. Therefore, the accumulation of PS in the cells, using the UCNPs as the drug carrier, would be considerably enhanced under the same incubation time. Most crucially, approximately 70% of the cells were killed under 980-nm laser irradiation, indicating the PDT efficiency was increased under NIR light. FCLA analysis revealed that the hybrid UCNPs exposed to the 660-nm laser induced higher ^1^O_2_ formation than the 980-nm laser did, because the photoexcitation of the Ce6 was less effective through the upconversion fluorescence emitted from the UCNPs (Figure 4b). However, the in vitro PDT experiments demonstrated a contrary result: NIR-laser irradiation provides more positive therapeutic outcomes in MCF-7 cell lines. Because the polycationic UCNPs improved the cellular uptake, the marked NIR-triggered PDT effect was due to longer laser wavelength and thus deeper tissue penetration. Although higher ^1^O_2_ was produced under red-light excitation in the tube test, NIR light could easily pass through the cell membrane and some organelles and reach the hybrid UCNPs. Therefore, surface Ce6 was effectively photoexcited under 980-nm laser irradiation, thus dramatically enhancing the overall PDT effect.

**Figure 6:**
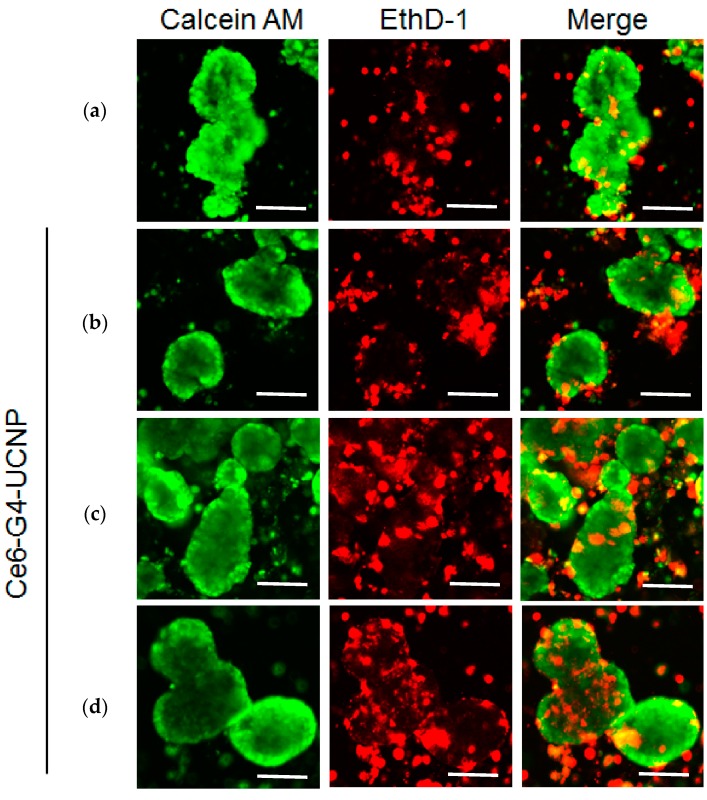

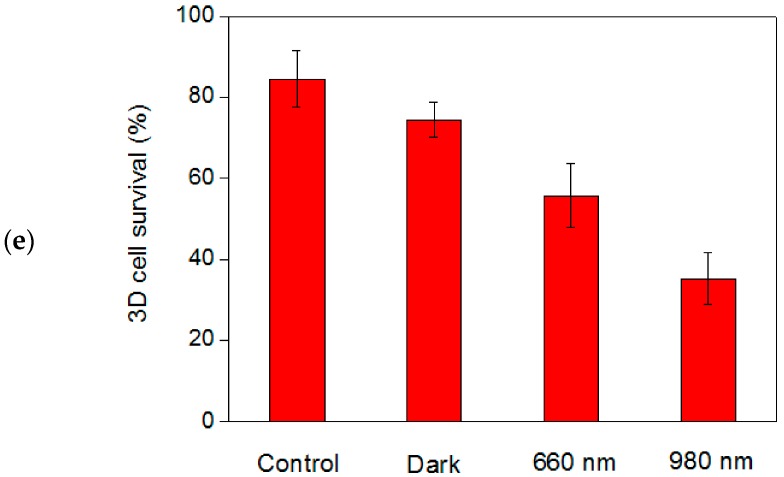
Fluorescence staining images for MCF-7 cells cultured in a 3D tumorsphere system: (**a**) control: live (calcein AM, green) and dead (EthD-1, red) cells in the absence of hybrid nanoparticles and light exposure; and (**b**–**d**) in the presence of hybrid nanoparticles in dark condition, and under 660-nm and 980-nm laser irradiation, respectively. (**e**) Quantitative analysis for the average distribution of live and dead cells in a tumorsphere with a standard deviation based on 10 spheres selected from the fluorescence images. The scale bar is 100 μm.

The MCF-7 cells were also cultured on ultralow attachment surfaces in serum-free mediums with limited growth factors to form the 3D scaffolds. These tumorspheres, which have been proven to possess CSC-like properties, served as an effective in vitro platform for screening anti-CSCs drugs. The 3D cells were also exposed to the 660-nm and 980-nm lasers to compare the wavelength-dependent PDT efficiencies. Immediately after exposure, live-dead staining was performed to evaluate viability distribution of the cells in tumorspheres [46]. Figure 6a shows the pretreatment distribution of the living and dead cells in the 3D spheres specifically labeled with green and red fluorophores. Additionally, Figure 6b demonstrates that after treating the cells with the hybrid UCNPs under darkness, the dead cells marked in red were mainly located at the peripheral of the 3D spheres. The natural apoptosis of the surrounding cells during the cultivation of the tumorspheres was the possible cause of this observation. When the treated 3D cells were exposed to the laser light, the dead portions were clearly located inside the spheres, indicating that the UCNPs successfully absorbed by the tumorspheres produced the PDT effect, not only at the surface but also at the center of spheres. Moreover, comparing Figure 6c,d, we noticed that the 980-nm laser induced higher cell death inside the spheres. This result indicates that the NIR-light penetrated the 3D scaffolds more deeply, thus triggering the PDT outcome according to the accumulated hybrid UCNPs. Furthermore, as shown in Figure 6e, the quantitative analysis was conducted by manually counting the live and dead cells, based on the spheres selected from the fluorescence images. The result was consistent with the survival test for 2D cells, confirming the highest PDT performance, the lowest percentage of cell survival under 980-nm laser irradiation. To our knowledge, this is the first example of NIR-light-driven PDT on a 3D tumorspherical platform using UCNPs as the PS, carrier, and activator, simultaneously.

**Figure 7:**
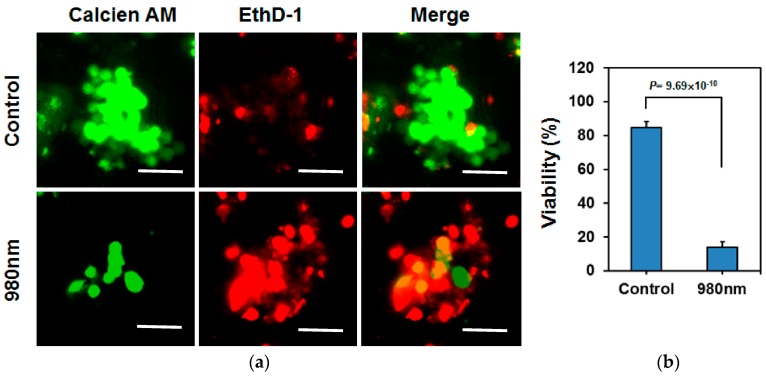
(**a**) Fluorescence staining images for live and dead MCF-7 cells in a tumorsphere in the presence of hybrid nanoparticles with higher loading capacity of Ce6 (1.36 μM). Control: live (calcein AM, green) and dead (EthD-1, red) cells in the absence of hybrid nanoparticles and light exposure. The scale bar is 50 μm. (**b**) The quantitative analysis based on 10 spheres selected from the images shows that less than 20% of cells survived under 980-nm laser irradiation for 20 min.

Tumorspheres driven from cancer cells have been proven to display characteristics of CSCs, which are considered the main cause of cancer recurrence. Because the 3D spheres grow divergently, from the core to peripheral, the cells inside the spheres may exhibit more characteristic CSC/progenitor cell properties than the cells located on the surface. For the MCF-7 cells cultured in the 3D tumorspheres, the hybrid UCNPs combined with a NIR laser exhibited remarkable deep-tissue PDT effects, allowing for more effective destruction of the CSCs located inside the spheres. Moreover, the PDT outcome can be readily enhanced by increasing the dosage of hybrid UCNPs, light exposure time, and the power of the incident laser. Figure 7 illustrates that the NIR-light-triggered PDT killed most of the cells in the tumorspheres when the exposure time was increased to 20 min under the treatment of the UCNPs with higher Ce6 loading (1.36 μM). Considering all aforementioned factors, hybrid UCNPs are promising NIR-triggered PDT modules for application in the treatment of spheroidal CSCs.

### 2.5. In Vivo Assessment for DNA Damage in Tumor Tissues

**Figure 8:**
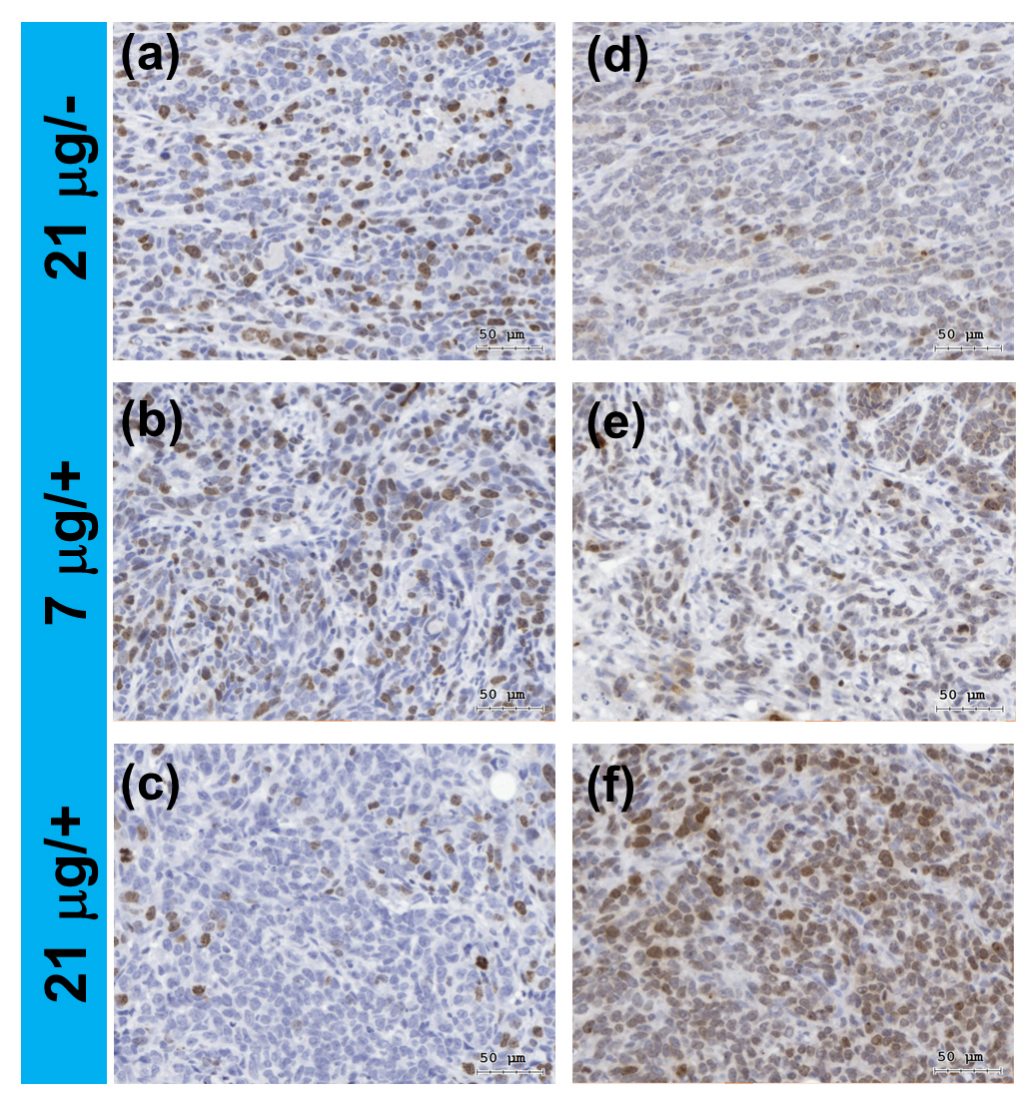
In vivo analysis for a murine 4T1 breast cancer model based on 980-nm laser light-trigged photodynamic therapy (PDT) using the hybrid nanoparticles as the photosensitizer. The tumors were treated with lower (7 μg) and higher (21 μg) dosage of the nanoparticles combined with (+) and without (−) light exposure. Immunohistochemical analysis of the tissue slices for: (**a**–**c**) Ki67; and (**d**–**f**) γH2AX^ser139^, corresponding to the protein markers for cell proliferation and DNA damage, respectively. The brown colors indicated positive staining for the markers. The cell nuclei were counterstained with hematoxylin (blue colors).

We further performed in vivo PDT analysis based on a 4T1 tumor-bearing mouse model treated with the hybrid UCNPs in combination with 980-nm laser exposure. The hybrid UCNPs with different dosages (7 μg and 21 μg) was loaded by intratumoral injection followed by exposure to laser for 10 min. From the immunohistochemical (IHC) analysis for the tissue slices, the expression of Ki67, a protein marker for cell proliferation [47], was obviously decreased in the tumor under higher dose combined with laser exposure (Figure 8a–c). Because of the observation of in vitro ^1^O_2_ production, which is a strong inducer for DNA damage including DNA strand breaks [48], we further examined whether DNA damage in tumors could be induced by the photosensitizing effect of the UCNPs with increasing dosage. The expression of γH2AX^ser139^, a protein marker for DNA double strand breaks [49], was massively induced under higher dose combined with laser exposure (Figure 8d–f). Moreover, based on the IHC images taken in four different fields, statistical analysis conducted by manually counting the cells indicates that the ratio of positively stained cells for the Ki67 marker decreased from 24.0% to 4.88% and for the γH2AX^ser139^ marker increased from 2.16% to 15.6% after laser exposure (Supplementary Materials, Figures S7 and S8). Accordingly, the preliminary data suggest that the tumor growth was effectively inhibited through the cellular DNA damage induced by the NIR-triggered PDT effect.

## 3. Conclusions

We successfully synthesized a dendrimer-functionalized hybrid UCNPs for performing NIR-light-driven PDT on human breast cancer cell lines and a murine breast cancer model. The water-soluble nanoparticles composed of lanthanide-doped NaYF_4_ nanocrystals encapsulated by the PAMAM dendrimers exhibited significant upconversion fluorescence under 980-nm laser irradiation. Thereby, the Ce6 molecules immobilized on the surface through an LBL strategy were effectively photoexcited to generate cytotoxic ROS. The chemiluminescence assay confirmed that ^1^O_2_ as the main ROS was readily accumulated during the photoexcitation and that the 660-nm LD was a more effective light source for photoexciting the hybrid UCNPs through the Q-band absorption of the Ce6; however, in vitro experiments revealed that the 980-nm-laser light had higher cytotoxicity in the MCF-7 cells cultured according to a conventional 2D model. The reverse PDT outcome was most likely due to the NIR light demonstrating higher penetration of a living system than visible light did. Moreover, compared with pristine Ce6 under visible-light excitation, the hybrid UCNPs displayed optimal in vitro PDT efficacy under NIR-light excitation. This finding is attributed to the enhancement of cellular uptake by the polycationic dendrimers through electrostatic interactions with polyanionic membrane lipids. Most notably, NIR light also induced an efficient PDT effect in the 3D cell model of MCF-7 tumorspheres, suggesting that hybrid UCNPs can be effectively applied in more complicated cellular textures. In vivo assessment based on immunohistochemical analysis for tumor tissues on a 4T1 tumor-bearing female mouse eventually shows significant inhibition of tumor cell proliferation through cellular DNA damage. Thus, the ^1^O_2_ produced from the hybrid UCNPs is the main cause for the successful in vitro and in vivo NIR-triggered PDT.

## 4. Materials and Methods

### 4.1. Materials and Instruments

The chemical reagents and organic solvents for materials synthesis were obtained as high-purity reagent-grade from commercial suppliers and used without further purification. The fourth generation PAMAM dendrimer (G4-NH_2_) was purchased from Dendritech, Inc. (Midland, MI, USA). For the materials analysis, FT-IR was performed on a Bruker Alpha FT spectrometer (Bruker Corp., Billerica, MA, USA). UV-Vis absorption spectra were recorded on a Thermo Genesys 10S UV-vis spectrometer (ThermoFisher Scientific, Waltham, MA, USA) equipped with a thermostatic cuvette holder. Fluorescence emission spectra for ^1^O_2_ analysis were recorded on a Hitachi F-2500 spectrometer (Hitachi High-Tech., Tokyo, Japan). TEM, EDXS, and XRD were performed on a Jeol JEM-2100 instrument, a Jeol JSM-6330F instrument (Jeol Ltd., Toyko, Japan), and a Bruker D2 PHASER X-ray Diffractometer (Bruker Corp., Billerica, MA, USA), respectively. Dynamic light scattering analysis involving particle size and zeta potential measurements was carried out by a Malvern Zetasizer Nano ZS (Malvern Instrument Ltd., Malvern, UK). TGA was recorded on a Thermo Cahn VersaTherm HS TG analyzer (ThermoFisher Scientific, Waltham, MA, USA). The analysis for upconversion fluorescence and photodynamic reaction were carried out on a homemade experimental apparatus using either 660-nm or 980-nm laser diode as the excitation source (output power: 2 W). The upconversion emission from the samples in a cuvette folder was collected and induced by a fiber bundle into a CCD imaging spectrometer (USB-4000, Ocean Optics, Largo, FL, USA) for spectra recording.

### 4.2. Synthesis of Hybrid UCNPs

NaYF_4_:Yb, Tm/NaYF_4_ UCNPs with a core-shell structure was synthesized by a solvothermal procedure [33]. In brief, for the synthesis of the core nanoparticles, anhydrous Y(CH_3_CO_2_)_3_ (372 mg), Yb(CH_3_CO_2_)_3_ (210 mg), and Tm(CH_3_CO_2_)_3_ (3.5 mg) were added to a 100 mL two-neck round-bottom flask containing oleic acid (35 mL). The solution was first purged with N_2_ and then heated slowly to 110 °C under reduced pressure until complete removal of residual water and oxygen. The temperature of the reaction flask was then lowered to 50 °C under a gentle N_2_ flow. During this time, an anhydrous methanol solution of ammonium fluoride (0.3 g) and sodium hydroxide (0.4 g) was prepared via sonication, and the mixture solution was then transferred to the two-neck flask via cannulation. The reaction flask was heated to 65 °C under reduced pressure until complete removal of methanol. Subsequently, the reaction temperature was increased to 300 °C as quick as possible and maintained at this temperature for 30 min under N_2_. After reaction, the nanoparticles were precipitated by adding excess ethanol (400 mL) and collected by centrifugation at 4500 rpm. The resulting pellet was redispersed in hexane and precipitated with excess ethanol. The dispersion-precipitation process was repeated until the removal of free oleic acids. The core-shell UCNPs with an outer layer of NaYF_4_ was synthesized following the same solvolthermal procedure except that the reaction time was increased to 90 min.

For the synthesis of citrate-modified UCNPs, 100 mg of core-shell UCNPs was dispersed in 60 mL of diethylene glycol (DEG) followed by adding 200 mg of trisodium citrate. The mixture was then heated to 200 °C with gentle stirring for 17 h. After cooling to room temperature, the modified UCNPs were collected by centrifugation under 12,000 rpm. The precipitate was rinsed with 20 mL of DEG for 3 times to remove nonadsorbed citrate ligands followed by rinsed with 20 mL of CH_2_Cl_2_ to remove DEG. The final product was obtained by drying under room temperature.

For the synthesis of dendrimer-encapsulated UCNPs via LBL method, 20 mg of citrate-modified UCNPs was dispersed in 15 mL of H_2_O followed by adding 40 μL of NH_2_-terminated PAMAM dendrimer (10 wt % in methanol). The mixture solution was then carefully adjusted to pH = 4 by adding HCl solution and allowed to stirring at room temperature for 24 h. After reaction, water was first removed by vacuum distillation, and the modified UCNPs was precipitated by adding 200 mL of cold acetone. The final product collected by centrifugation under 12,000 rpm was drying under vacuum under the temperature less than 35 °C.

For the synthesis of hybrid UCNPs, 10 mg of the dendrimer-encapsulated UCNPs was dispersed in 5 mL of methanol with gentle stirring followed by adding Ce6 stock solution (1.5 × 10^−3^ M in methanol). The feeding amount of Ce6 was fine adjusted by controlling the injection volume of the stock solution. The mixture solution was stirred under room temperature for 2 h, and the solvent was removed under reduced pressure. The crude UCNPs was rinsed with CH_2_Cl_2_ to wash off the nonadsorbed Ce6 until the CH_2_Cl_2_ layer was colorless. The final hybrid nanoparticles was drying under room temperature, and the loading capacity of the Ce6 was determined by fluorescence spectroscopy analysis. Prior to biological experiments, the hybrid UCNPs was prepared in stock aqueous solutions with corresponding Ce6 concentrations of 0.43, 2.50 and 13.6 μM.

### 4.3. The Singlet Oxygen Assay

In a typical FCLA experiment, 20 μL of a FCLA/ethanol solution (0.7 mg/mL) was added to 2 mL of a UCNPs solution and transferred into a 1-cm quartz cuvette. The solution was irradiated with an either 660-nm or 980-nm laser diode, and the emission intensity of the exposed solutions at 519-nm was recorded every 2 min under 496-nm excitation by fluorescence spectroscopy. For the control experiment, FCLA emission was also recorded at the same conditions in the absence of laser irradiation.

### 4.4. Cell Culture and Tumorsphere Cultivation

For a conventional 2D cell model, MCF-7 cells were obtained from American Type Culture Collection (Manassas, VA, USA) and cultured in DMEM medium (Invitrogen Corporation, Grand Island, NY, USA) containing 10% fetal bovine serum (Invitrogen) and 5 μg/mL insulin (Sigma-Aldrich, St. Louis, MO, USA). For a 3D tumorsphere cultivation, MCF-7 cells were prepared as a density of 1 × 10^4^ cells/mL in DMEM/F12 medium (Invitrogen Corporation) containing 0.5% methylcellulose (Sigma-Aldrich), 0.4% bovine serum albumin (Sigma-Aldrich), 10 ng/mL of EGF (PeproTech, Rocky Hill, NJ, USA), 10 ng/mL bFGF (PeproTech, Rocky Hill, NJ, USA), 5 μg/mL insulin, 1 μM hydrocortisone (Sigma-Aldrich) and 4 μg/mL heparin (Sigma-Aldrich). Cell suspension (2 mL) was seeded into wells of suspension culture 6-well-plate (Greiner Bio One International GmbH, Frickenhausen, Germany) and incubated for 7 days.

### 4.5. Cell Viability Assay

To determine the PDT efficiency, cell proliferation for the cells cultured in a 2D model after light exposure were determined by CCK8 cell viability assay reagent (Sigma-Aldrich) using a microplate reader (Molecular Devices, Sunnyvale, CA, USA) according to the manufacturer’s recommendations. For the in vitro photoexicitation experiment, MCF-7 cells were firstly suspended as 1 × 10^5^ per mL for 2.5 mL and transferred into a 1-cm quartz cuvette and directly exposed to a laser beam with either 660-nm or 980-nm wavelength at fixed time intervals under gentle agitation. The exposed cells were then seeded into 96-well-plate at 1 × 10^4^ per well and cultured for another 48 h. The absorbance values at 440 nm of non-treatment control were set as 100% of cell proliferation. Cell proliferation for the cells cultivated in a 3D model was determined by Live/Dead cell viability assay (Thermo Fisher Scientific, Waltham, MA, USA). The viable or dead cells were specifically stained by green and red fluorophores derived from calcein AM and ethidium homodimer-1, respectively. Following the same protocol of the in vitro photoexcitation, the exposed tumorspheres were collected and washed in phosphate buffered saline (PBS) and then suspended in 250 μL PBS containing 4 μM of calcien AM/2 μM of ethidium homodimer-1. After incubation at 37 °C for 30 min, fluorescence images with respect to the distributions of viable and dead cells in tumorspheres were captured by inverted fluorescence microscopy (AE30, Motic Electric Group Co., Ltd., Xiamen, China) and counted with ImageJ software (NIH, Bethesda, MD, USA).

### 4.6. In Vivo Assessment for Tumor Tissues

Murine 4T1 breast cancer cells were obtained from American Type Culture Collection (ATCC, Manassas, VA, USA) and maintained by DMEM containing 10% fetal bovine serum. The orthotopic breast cancer model was conducted by injection of 4T1 cells into mammary fat pads of BALB/c female mice (purchased from the National Laboratory Animal Center of Taiwan, Taipei, Taiwan) according to the previous report [50]. All the animal studies were operated following a protocol approved by Institutional Animal Care & Utilization Committee of Chung Shan Medical University (IACUC Approval No. 1379). Briefly, 2 × 10^5^ 4T1 cells were suspended in 50 μL matrigel (BD Biosciences, San Jose, CA, USA) at a concentration of 2 mg/mL and injected the 4th pair of mammary fat pad. The treatment was begun when tumor volume reached to 100 mm^3^ (around 21 days after injection). The tumors were exposed with 980-nm laser after intratumoral injection of the hybrid UCNPs for 10 min per time and the treatment was performed every 3 days for total 3 times. Three mice were sacrificed at day 3 after the last treatment and tumors were taken out for performing immunohistochemistrial analysis. The tumors were fixed with 3.7% formaldehyde and embedded in paraffin. Four micrograms of paraffin sections were sliced followed by dewaxed/rehydration steps and used for detection of Ki-67 (a marker for cell proliferation) and γH2AX^ser139^ (a marker for DNA double strand breaks) expression. Briefly, the sections were incubated with monoclonal rabbit anti-Ki67 (Cat. No. GTX16667, GeneTex International Corporation, Hsinchu City, Taiwan) or monoclonal rabbit anti-γH2AXser139 (Cat. No. NB100-79967, Novus Biologicals, Littleton, CO, USA) using a standard avidin-biotin-peroxidase complex method. 3,3′-Diaminobenzidine (DAKO, Carpinteria, CA, USA) was then used to detect the positive staining.

## Supplementary Materials

The following are available online at http://www.mdpi.com/2079-4991/7/9/269/s1, Figure S1: XRD analysis of: (**a**) core-shell; and (**b**) core NaYF_4_ nanocrystals with α-phase (cubic) pattern; Figure S2: EDXS analysis for the NaYF_4_ nanocrustals with successful Yb and Tm-doping; Figure S3: TGA analysis for: (**a**) ligand-free; (**b**) oleate; and (**c**) citrate-modified upconversion nanoparticles (UCNPs); Figure S4: zeta potential measurements for UCNPs sequentially encapsulated with: (**a**) citrate; (**b**) PAMAM dendrimer; and (**c**) chlorin e6 (Ce6); Figure S5: FT-IR analysis for: (**a**) citrate; and (**b**) PAMAM dendrimer-modified UCNPs. The arrows indicate the characteristic absorption bands of the surface functional groups; Figure S6: particle size distribution for PAMAM dendrimer-modified UCNPs; Figure S7: the Immunohistochemical (IHC) analysis of the tissue slices for Ki67 protein marker in four different fields. The tumors were treated with lower (7 μg) and higher (21 μg) dosage of the UCNPs combined with (+) and without (−) 980-m laser exposure: (**a**) 21 μg/−; (**b**) 7 μg/+; and (**c**) 21 μg/+; and (**d**) the ratios of the positively stained cells for (a–c) analyzed by manually counting the cells in the IHC images; Figure S8: the Immunohistochemical (IHC) analysis of the tissue slices for γH2AX^ser139^ protein marker in four different fields. The tumors were treated with lower (7 μg) and higher (21 μg) dosage of the UCNPs combined with (+) and without (−) 980-m laser exposure: (**a**) 21 μg/−; (**b**) 7 μg/+; and (**c**) 21 μg/+; and (**d**) the ratios of the positively stained cells for (a–c) analyzed by manually counting the cells in the IHC images.
