# Supplementary material for: Near-Infrared-Triggered Photodynamic Therapy toward Breast Cancer Cells Using Dendrimer-Functionalized Upconversion Nanoparticles
Source: Nanomaterials (Basel). 2017 Sep 11;7(9):269. doi: 10.3390/nano7090269 (PMC5618380; doi:10.3390/nano7090269)
Supplement: Supplementary file 1 [file nanomaterials-07-00269-s001.docx]

**Supporting information**

**Near-infrared-triggered photodynamic therapy toward breast cancer cells using dendrimer-functionalized upconversion nanoparticles**

Bing-Yen Wang ^1,2,3,4^, Ming-Liang Liao ^5^, Guan-Ci Hong ^6^, Wen-Wei Chang ^6,^*, and

Chih-Chien Chu ^5,7,^*

^1^ Division of Thoracic Surgery, Department of Surgery, Changhua Christian Hospital and Chung Shan Medical University, Taichung, Taiwan; [156283@cch.org.tw](mailto:156283@cch.org.tw)

^2^ School of Medicine, Chung Shan Medical University, Taichung, Taiwan

^3^ Institute of Genomics and Bioinformatics, National Chung Hsing University, Taichung, Taiwan

^4^ School of Medicine, Kaohsiung Medical University, Kaohsiung, Taiwan

^5^ Department of Medical Applied Chemistry, Chung Shan Medical University, Taichung, Taiwan; [withoutyou@hotmail.com.tw](mailto:withoutyou@hotmail.com.tw)

^6^ Department of Biomedical Sciences, Chung Shan Medical University, Taichung, Taiwan; [gqttw981@mail.ntust.edu.tw](mailto:gqttw981@mail.ntust.edu.tw)

^7^ Department of Medical Education, Chung Shan Medical University Hospital, Taichung, Taiwan

*Correspondence: changww@csmu.edu.tw (W.-W.C.); jrchu@csmu.edu.tw (C.-C.C.);
Tel.: +886-4-2473-0022-12317 (W.-W.C.); +886-4-2473-0022-12227 (C.-C.C.)

**Fig. S1**. XRD analysis of (a) core-shell and (b) core NaYF_4_ nanocrystals with α–phase (cubic) pattern.





| Element | Atomic % |
| --- | --- |
| F | 52 |
| Na | 14 |
| Y | 29 |
| Tm, Yb | 5 |

**Fig. S2**. EDXS analysis for the NaYF_4_ nanocrustals with successful Yb and Tm-doping.

**Fig. S3**. TGA analysis for (a) ligand-free, (b) oleate, and (c) citrate-modified upconversion nanoparticles (UCNPs).


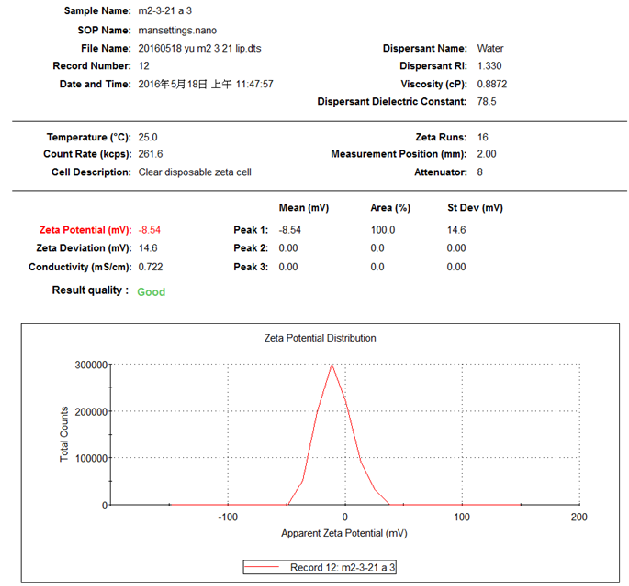


**(a)**


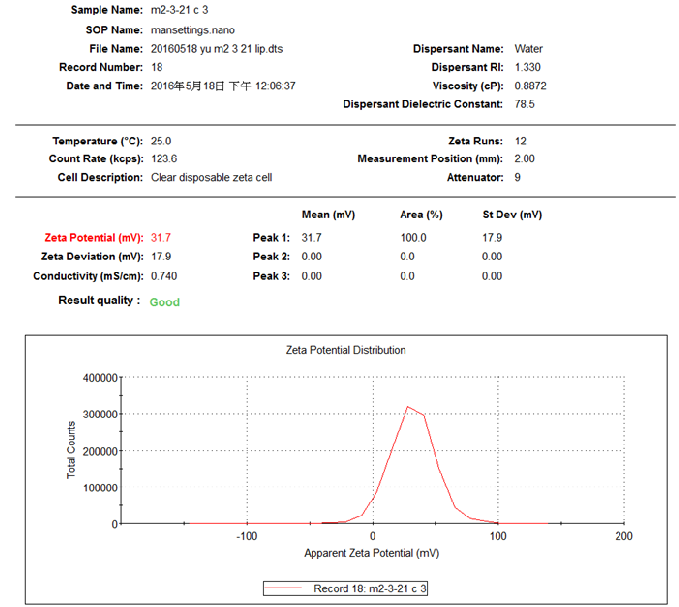


**(b)**


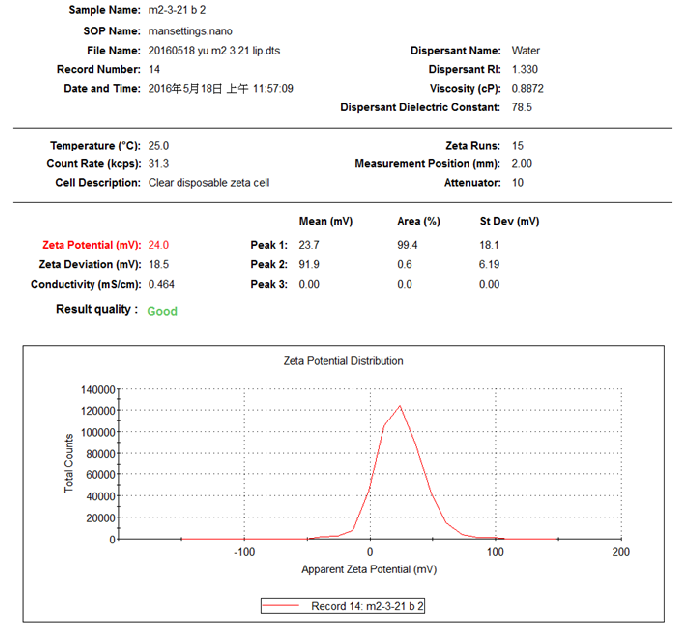


**(c)**

**Fig. S4**. Zeta potential measurements for UCNPs sequentially encapsulated with (**a**) citrate, **(b**) PAMAM dendrimer, and (**c**) chlorin e6 (Ce6).

**Fig. S5**. FT-IR analysis for (a) citrate and (b) PAMAM dendrimer-modified UCNPs. The arrows indicate the characteristic absorption bands of the surface functional groups.


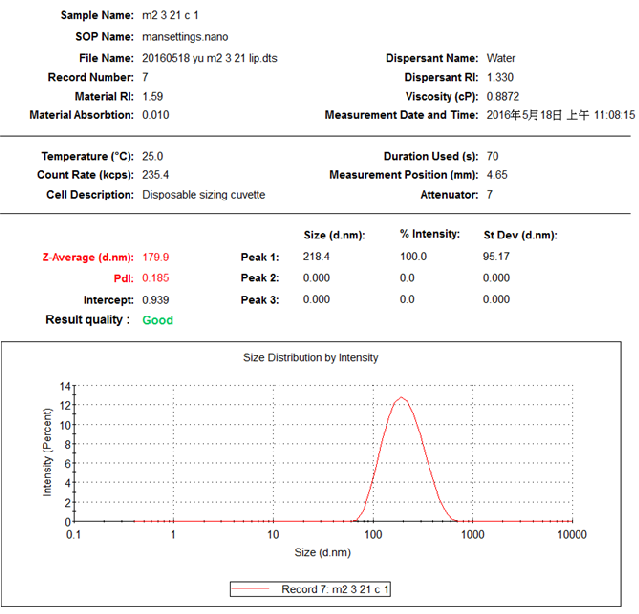


**Fig. S6**. Particle size distribution for PAMAM dendrimer-modified UCNPs.


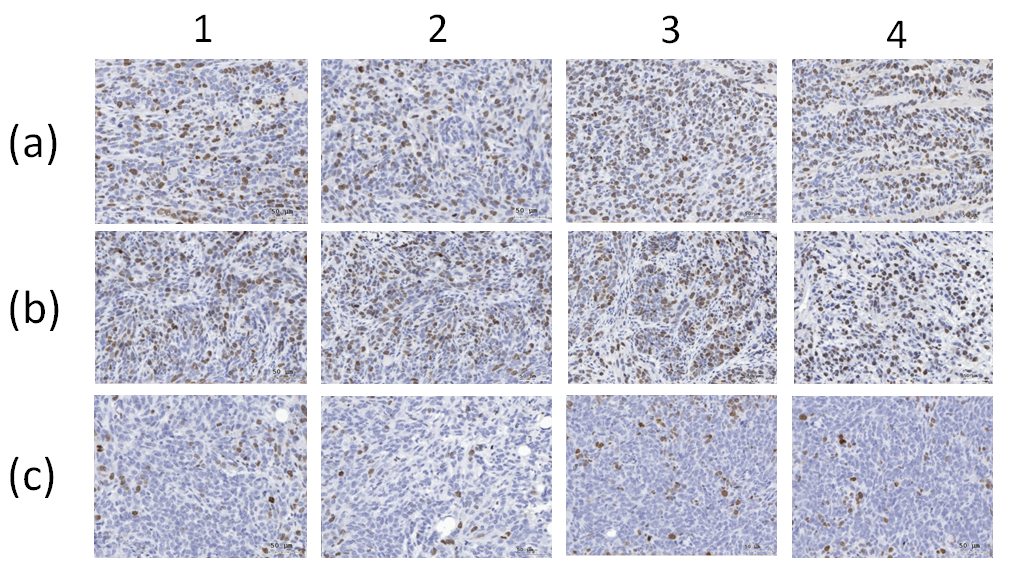


(d)


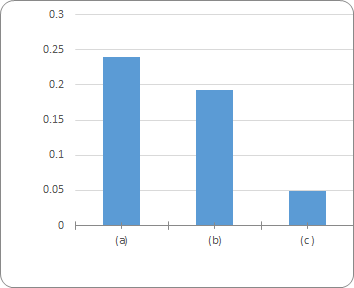


4.88%

19.3%

24.0%

**Fig. S7**. The Immunohistochemical (IHC) analysis of the tissue slices for Ki67 protein marker in four different fields. The tumors were treated with lower (7 μg) and higher (21 μg) dosage of the UCNPs combined with (+) and without (−) 980-m laser exposure: (**a**) 21 μg/−; (**b**) 7 μg/+; (**c**) 21 μg/+. (**d**) The ratios of the positively stained cells for (**a**), (**b**), and (**c**) analyzed by manually counting the cells in the IHC images.


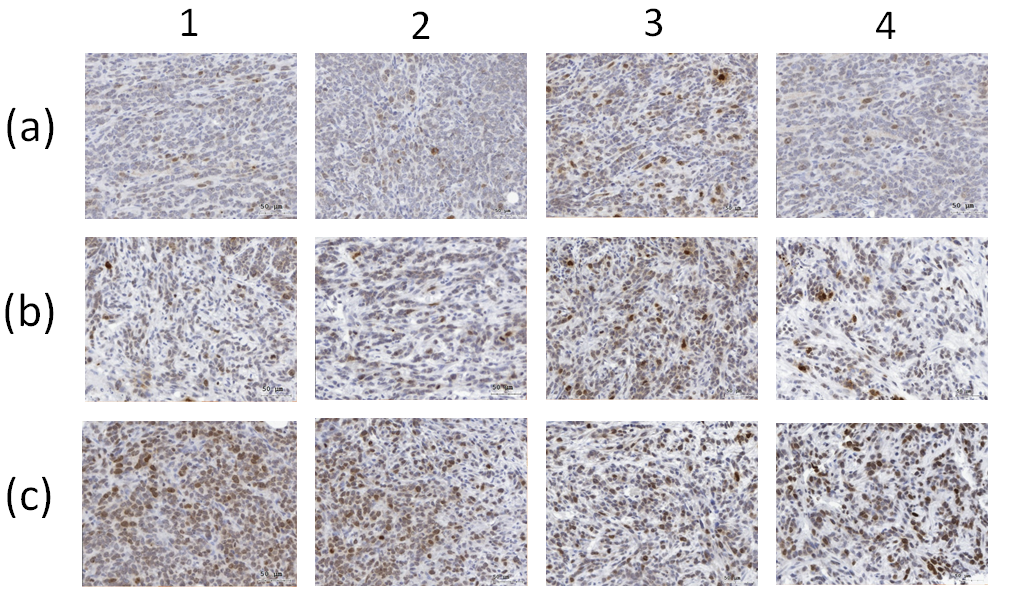


(d)


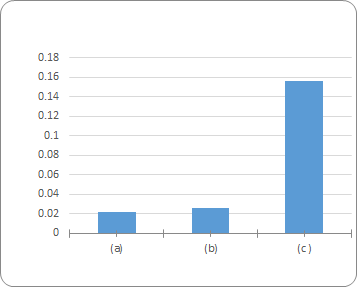


15.6%

2.55%

2.16%

**Fig. S8**. The Immunohistochemical (IHC) analysis of the tissue slices for γH2AX^ser139^ protein marker in four different fields. The tumors were treated with lower (7 μg) and higher (21 μg) dosage of the UCNPs combined with (+) and without (−) 980-m laser exposure: (**a**) 21 μg/−; (**b**) 7 μg/+; (**c**) 21 μg/+. (**d**) The ratios of the positively stained cells for (**a**), (**b**), and (**c**) analyzed by manually counting the cells in the IHC images.
